# Prognostic Impact of Transient Return of Spontaneous Circulation According to Low-Flow Time in Patients with Out-of-Hospital Cardiac Arrest Undergoing Extracorporeal Cardiopulmonary Resuscitation: A Nationwide Cohort Study

**DOI:** 10.3390/jcm15197354

**Published:** 2026-09-22

**Authors:** Seongjin Noh, Jae-Guk Kim, Gu-Hyun Kang, Yong-Soo Jang, Wonhee Kim, Hyun-Young Choi

**Affiliations:** Department of Emergency Medicine, Kangnam Sacred Heart Hospital, Hallym University College of Medicine, Seoul 07441, Republic of Korea; axcee@naver.com (S.N.); emkang@hallym.or.kr (G.-H.K.); amicoys@hallym.or.kr (Y.-S.J.); wonsee02@hallym.or.kr (W.K.); chy6049@hallym.or.kr (H.-Y.C.)

**Keywords:** out-of-hospital cardiac arrest, extracorporeal cardiopulmonary resuscitation, transient return of spontaneous circulation, low-flow time

## Abstract

**Background/Objectives:** Low-flow time (LFT) is a major prognostic factor in patients undergoing extracorporeal cardiopulmonary resuscitation (ECPR), but chronological LFT may not fully reflect ischemic burden when transient return of spontaneous circulation (ROSC) occurs. We investigated whether the association between transient ROSC and outcomes varies according to LFT. **Methods**: This nationwide retrospective cohort study included 810 adults with witnessed out-of-hospital cardiac arrest who underwent ECPR. The primary outcome was survival to hospital discharge, and the secondary outcome was good neurological outcome. A prespecified subgroup analysis compared LFT ≤90 versus >90 min, followed by an exploratory analysis using <60, 60–90, and >90 min categories. Multivariable logistic regression and interaction analyses were performed. **Results:** Among 810 patients, 450 (55.6%) experienced transient ROSC. In the prespecified analysis, transient ROSC was associated with survival in patients with LFT ≤90 min (adjusted odds ratio [AOR], 2.375; 95% confidence interval [CI], 1.076–5.241; *p* = 0.032), but not in those with LFT >90 min (AOR, 0.884; 95% CI, 0.595–1.315; *p* = 0.543), with a significant interaction (*p* = 0.036). The exploratory three-category analysis showed a significant association in the 60–90-min subgroup (AOR, 3.399; 95% CI, 1.021–11.316; *p* = 0.046), although the global interaction was not significant (*p* = 0.126). No significant interaction was observed for good neurological outcome. The prespecified survival interaction remained significant in sensitivity analyses. **Conclusions:** The association between transient ROSC and survival after ECPR differed according to LFT using the prespecified 90-min cutoff. Transient ROSC may provide prognostic information complementary to chronological LFT, whereas the three-category findings should be considered exploratory.

## 1. Introduction

Despite substantial improvements in resuscitation strategies and post-resuscitation care, out-of-hospital cardiac arrest (OHCA) continues to be associated with high mortality and considerable neurological morbidity. For patients with refractory OHCA who do not achieve sustained return of spontaneous circulation (ROSC) despite conventional cardiopulmonary resuscitation (CPR), extracorporeal cardiopulmonary resuscitation (ECPR) provides an alternative means of re-establishing systemic perfusion [1,2]. However, ECPR outcomes remain highly variable, emphasizing the importance of identifying prognostic factors that can guide patient selection and clinical decision-making [1,2,3].

Among these factors, low-flow time (LFT) is one of the strongest determinants of ECPR outcome [4,5,6]. LFT, typically measured from the start of CPR to the initiation of extracorporeal blood flow, represents the period during which systemic and cerebral perfusion remains critically inadequate, reflects the duration of severely compromised systemic and cerebral perfusion [3,4]. Previous studies have consistently shown that prolonged LFT is associated with decreased survival and poor neurological outcomes after ECPR [3,4,5,6]. In particular, outcomes deteriorate substantially as LFT extends beyond approximately 40–60 min, highlighting the importance of minimizing the duration of low-flow circulation [2,3,4,5].

However, chronological LFT may not fully represent the physiological ischemic burden during resuscitation, since some patients undergoing ECPR experience transient ROSC before extracorporeal circulation is established [7,8]. Although these patients may have the same chronological LFT as those without transient ROSC, temporary restoration of spontaneous circulation may interrupt otherwise continuous exposure to severely compromised perfusion. Transient ROSC may also reflect greater cardiovascular reserve, reversibility of the underlying cause of cardiac arrest, or responsiveness to resuscitative interventions. Therefore, its prognostic significance may vary by duration of low-flow exposure [7,8,9].

Previous studies have shown good associations between transient ROSC and outcomes after ECPR. A multicentre Japanese study showed that transient ROSC before ECPR was associated with good neurological outcome and survival [8], and a subsequent study of patients with prolonged LFT ≥ 60 min identified transient ROSC as an independent predictor of good neurological recovery [7]. Conversely, findings from our previous nationwide Korean cohort demonstrated that transient ROSC was not significantly associated with survival to hospital discharge or good neurological outcomes among patients undergoing ECPR [9]. However, whether the prognostic significance of transient ROSC differs by LFT remains unclear.

Because chronological LFT does not account for transient ROSC, it may not fully reflect the actual ischemic burden in patients experiencing it. We investigated whether the association between transient ROSC before ECPR and clinical outcomes varied by LFT in adult patients with witnessed OHCA undergoing ECPR for refractory cardiac arrest.

## 2. Materials and Methods

### 2.1. Study Design and Data Source

We performed a nationwide retrospective cohort study using data obtained from the Korean Out-of-Hospital Cardiac Arrest Surveillance (OHCAS) registry. The OHCAS registry includes patients with OHCA transported by emergency medical services (EMS) and contains information regarding prehospital resuscitation, emergency department (ED) management, in-hospital treatment, and clinical outcomes. The registry is maintained by the Korea Disease Control and Prevention Agency (KDCA), and data are collected and reviewed using a standardized surveillance protocol.

Several physiological and resuscitation-related variables of relevance to this study were unavailable in the registry. These included hemodynamic data during and following ROSC, laboratory measurements such as serum lactate levels and arterial blood gas values, and specific features of transient ROSC episodes, including their duration, frequency, timing, and associated hemodynamic status. Therefore, these variables could not be incorporated into the present analysis.

The Institutional Review Board of Kangnam Sacred Heart Hospital approved the study protocol (HKS 2026-04-029) and granted a waiver of informed consent because the analysis was based on de-identified registry data. The KDCA authorized the use of the OHCAS database in 2026. Reporting followed the Strengthening the Reporting of Observational Studies in Epidemiology (STROBE) guidelines.

### 2.2. Study Population

Cases recorded in the OHCAS registry between January 2016 and December 2024 were identified, yielding a total of 284,608 patients with OHCA. The study population was restricted to adult patients with witnessed OHCA of presumed medical aetiology who received ECPR in the ED for refractory cardiac arrest.

We excluded patients aged <18 years; those with unwitnessed cardiac arrest; those with cardiac arrest due to non-medical causes, including trauma, drowning, or intoxication; those who achieved sustained ROSC before ECPR initiation; those with documented do-not-resuscitate orders; those declared dead on hospital arrival; those who did not undergo ECPR; those with missing LFT or outcome data; and those with incomplete ECPR. After applying the eligibility criteria, 810 patients were included in the final analysis.

No upper age limit was applied because age was not used as an absolute exclusion criterion in this registry-based study [10,11]. ECPR was performed among selected patients with refractory OHCA according to institutional protocols and the clinical judgment of the treating physicians. Because the OHCAS registry does not provide detailed information regarding institution-specific ECPR eligibility criteria or the reasons for withholding ECPR in individual patients, these factors could not be further evaluated.

Patients were categorized according to whether transient ROSC occurred before ECPR initiation, forming the transient ROSC and non-transient ROSC groups.

ECPR procedural outcomes were classified as successfully completed, incompletely terminated, or unknown. Successfully completed ECPR was defined as successful establishment of extracorporeal circulation with ECMO flow. Incomplete ECPR was defined as an attempted procedure that was terminated before successful ECMO flow was established because of unexpected procedural complications or deterioration in the patient’s clinical condition. Cases with an unknown procedural outcome, in which ECPR initiation was documented but its completion could not be confirmed because of missing or lost records, were classified as not having undergone ECPR. Only patients with successfully completed ECPR were included in the final analysis.

### 2.3. Definitions and Data Collection

Demographic and resuscitation-related variables included age, sex, bystander CPR, presumed cause of cardiac arrest, initial cardiac rhythm, pre-existing comorbidity, use of mechanical CPR, post-cardiac arrest care, and LFT. Because the study population was restricted to patients with witnessed OHCA, witnessed arrest status was not included as an explanatory variable in subsequent regression analyses.

An initial rhythm of ventricular fibrillation or pulseless ventricular tachycardia was considered shockable. Pre-existing comorbid conditions assessed in this study comprised hypertension, diabetes mellitus, heart disease, chronic kidney disease, stroke, and respiratory disease, as detailed in Table S1. Post-cardiac arrest care variables included reperfusion therapy and targeted temperature management (TTM). Reperfusion therapy was defined as intravenous thrombolysis or percutaneous coronary intervention.

Transient ROSC was considered present when spontaneous circulation, confirmed by a palpable pulse or measurable blood pressure, was maintained for more than 1 min but less than 20 min before the initiation of ECPR. Such episodes could occur either before or after hospital arrival, although the registry did not reliably capture the specific setting in which each episode occurred. ROSC was evaluated during routine resuscitation by EMS personnel in the prehospital setting and by the treating physicians following hospital arrival. The registry recorded the occurrence of transient ROSC but did not provide sufficiently detailed information regarding the exact duration, number, timing, or hemodynamic quality of individual transient ROSC episodes.

LFT was defined as the chronological interval from the initiation of CPR to the establishment of extracorporeal blood flow. Episodes of transient ROSC occurring during this interval were not subtracted from LFT. Thus, LFT represented chronological elapsed time rather than the cumulative duration of actual low-flow circulation. Episodes of transient ROSC before ECPR initiation were not considered termination of the low-flow interval [12]. Accordingly, LFT in the present study represented the chronological interval between CPR initiation and establishment of ECMO flow rather than the cumulative duration of CPR without spontaneous circulation.

Neurological outcome at hospital discharge was evaluated using the Cerebral Performance Category (CPC) scale. CPC scores of 1–2 were classified as a good neurological outcome, whereas scores of 3–5 were classified as a poor neurological outcome (Table S2).

### 2.4. Outcome Measures

The primary outcome was survival to hospital discharge. The secondary outcome was good neurological outcome at hospital discharge, defined as a CPC score of 1 or 2.

### 2.5. Statistical Analysis

Baseline characteristics were compared between patients with and without transient ROSC before ECPR initiation. Categorical data are summarized as frequencies and percentages and were compared using Pearson’s chi-square or Fisher’s exact test, as appropriate. Continuous data are expressed as mean ± standard deviation or median (interquartile range), depending on their distribution, and were compared using the independent-samples *t*-test or Mann–Whitney U test, respectively. Missing data were handled using complete-case analysis because the proportion of missing observations among the variables included in the analyses was <5% [13,14] The extent of missing data for each variable is presented Table S6.

Multivariable logistic regression was performed to evaluate the association between transient ROSC and clinical outcomes in the overall study population. Survival to hospital discharge and good neurological outcome were analysed separately as dependent variables, with transient ROSC as the primary exposure of interest. Covariates were selected a priori based on clinical relevance and previously reported associations with outcomes after cardiac arrest and ECPR. Variables prespecified for adjustment were age, sex, bystander CPR, presumed cardiac origin, initial shockable rhythm, hypertension, diabetes mellitus, heart disease, chronic kidney disease, stroke, respiratory disease, mechanical CPR, reperfusion therapy, TTM, and LFT. Witnessed arrest was not included as a covariate because the study population was restricted to patients with witnessed cardiac arrest. Adjusted odds ratios (AORs) and corresponding 95% confidence intervals (CIs) were calculated.

Covariates for the primary multivariable models were prespecified based on clinical relevance and included variables available before or during the resuscitation phase: age, sex, bystander CPR, cause of cardiac arrest, initial shockable rhythm, hypertension, diabetes mellitus, heart disease, chronic kidney disease, stroke, respiratory disease, mechanical CPR, and LFT. All covariates were simultaneously entered into the models using the enter method. Post-arrest treatments, including reperfusion therapy and targeted temperature management (TTM), were not included in the primary models because these interventions occurred after the exposure and their inclusion could alter the interpretation of the estimated association between transient ROSC and clinical outcomes. As a sensitivity analysis, multivariable logistic regression models were additionally adjusted for reperfusion therapy and TTM to evaluate the robustness of the primary findings to adjustment for post-arrest treatments. The same subgroup and interaction analyses were repeated using these expanded models.

To investigate whether the association between transient ROSC and clinical outcomes varied by low-flow exposure duration, subgroup analysis was performed. The primary analysis used a prespecified low-flow time cut-off of 90 min (≤90 min vs. >90 min), based on prior clinical literature and commonly used ECPR eligibility criteria. A secondary exploratory analysis categorized low-flow time as <60 min, 60–90 min, and >90 min. Given the exploratory nature of this analysis, subgroup-specific findings were interpreted as hypothesis-generating.

The 90-min cutoff was used to distinguish prolonged from markedly prolonged low-flow exposure, based on previous observational studies that good outcomes may still occur despite prolonged LFT but become increasingly uncommon with very prolonged resuscitation [7,15]. Exploratory subgroup analysis was performed by further stratifying patients into three LFT categories (<60 min, 60–90 min, and >90 min) to characterize the association between transient ROSC and outcomes across different durations of low-flow exposure. The 60-min threshold was selected because contemporary ECPR recommendations emphasize the establishment of extracorporeal circulation within approximately 60 min of cardiac arrest whenever feasible [3]. This additional stratification was used to distinguish relatively shorter LFT from intermediate and markedly prolonged LFT and to examine whether the association between transient ROSC and outcomes differed across these clinically relevant intervals. Multivariable logistic regression was then performed within each LFT subgroup using the enter method to evaluate the association of transient ROSC with survival to hospital discharge and good neurological outcome. All prespecified covariates were simultaneously entered into each model, including LFT as a continuous covariate to account for residual variation in low-flow duration within each category.

Formal interaction analyses were performed in the full cohort to assess whether LFT modified the association between transient ROSC and clinical outcomes. Interaction terms between transient ROSC and LFT category were incorporated into multivariable logistic regression models. For the dichotomized LFT analysis (≤90 vs. >90 min), a single transient ROSC × LFT category interaction term was evaluated. For the three-category analysis (<60, 60–90, and >90 min), interaction terms were constructed using indicator variables for LFT categories, and the overall interaction was assessed by jointly testing these terms using a likelihood-ratio test comparing models with and without the interaction terms. Interaction analyses were conducted separately for survival to hospital discharge and good neurological outcome.

To address the potential for model overfitting due to the limited number of outcome events in the shorter LFT subgroups, an additional sensitivity analysis was performed using a reduced multivariable logistic regression model. The enter method was retained, with seven clinically important covariates available before or during resuscitation simultaneously entered into the model: age, sex, bystander CPR, initial shockable rhythm, cardiac cause, mechanical CPR, and continuous LFT. These covariates were selected based on their clinical relevance rather than statistical significance in the study dataset. The subgroup and interaction analyses were repeated using this reduced adjustment model to assess the robustness of the primary findings to a more parsimonious model specification.

A two-sided *p* value of <0.05 was considered statistically significant. All statistical analyses were performed using IBM SPSS Statistics version 26.0 (IBM Corp., Armonk, NY, USA) and R version 3.3.2 (R Foundation for Statistical Computing, Vienna, Austria).

## 3. Results

### 3.1. Study Population and Baseline Characteristics

During the study period, 283,608 patients with EMS-treated OHCA were identified. After applying predefined exclusion criteria, 810 adult patients with witnessed OHCA who underwent ECPR in the ED were included in the final analysis (Figure 1). Of these, 360 (44.4%) did not experience transient ROSC before ECPR initiation, whereas 450 (55.6%) experienced transient ROSC.

Table S3 summarizes the baseline characteristics of the 810 patients stratified by the presence or absence of transient ROSC before ECPR initiation. The overall cohort had a median age of 56.0 years (IQR, 45.0–64.0) and most patients were male (83.2%). Bystander CPR was performed in 42.1%, the presumed cause of arrest was cardiac in 96.5%, and an initial shockable rhythm was reported in 37.7%. Demographics, cardiac arrest characteristics, pre-existing comorbidities, and use of mechanical CPR and post-arrest treatments were comparable between patients with and without transient ROSC, with no significant differences observed (all *p* > 0.05). Similar findings were observed for reperfusion therapy (71.6% vs. 67.5%), TTM use (18.0% vs. 18.3%), and median LFT (108.0 [IQR, 91.0–140.0] vs. 110.0 [IQR, 95.0–153.5] min).

Overall, 184 patients (22.7%) survived to hospital discharge, and 122 (15.1%) had a good neurological outcome. In the unadjusted comparison, the rates of these outcomes were higher among patients with transient ROSC than among those without, but the differences were small and did not reach statistical significance (23.3% vs. 21.9% for survival; 15.6% vs. 14.4% for neurological outcome; all *p* > 0.05).

### 3.2. Association Between Transient ROSC and Clinical Outcomes in the Overall Cohort

In the overall study population, after multivariable adjustment, transient ROSC was not significantly associated with survival to hospital discharge (AOR, 1.025; 95% CI, 0.717–1.464; *p* = 0.892) or good neurological outcome at hospital discharge (AOR, 1.016; 95% CI, 0.668–1.544; *p* = 0.941) (Table S4).

### 3.3. Study Population According to Low-Flow Time Subgroups

Demographic characteristics, arrest-related factors, pre-existing comorbidities, post-cardiac arrest treatments, and LFT were generally comparable between patients with and without transient ROSC across the LFT subgroups. In the LFT <60-min subgroup, no significant differences in the evaluated baseline characteristics were observed between the two groups (all *p* > 0.05). Survival to hospital discharge was numerically higher in patients with transient ROSC than in those without transient ROSC (37.5% vs. 19.4%, *p* = 0.162), whereas the rates of good neurological outcome were similar between the groups (20.0% vs. 16.1%, *p* = 0.913) (Table 1). In the LFT 60–90-min subgroup, mechanical CPR was more frequently used in patients with transient ROSC than in those without transient ROSC (44.6% vs. 18.4%, *p* = 0.013), whereas no significant differences were observed in the other baseline characteristics. Survival to hospital discharge was 32.3% in patients with transient ROSC and 18.4% in those without transient ROSC (*p* = 0.194), while good neurological outcome occurred in 16.9% and 13.2%, respectively (*p* = 0.820) (Table 2). A similar difference in mechanical CPR use was observed in the combined LFT ≤90-min subgroup (41.9% vs. 24.6%, *p* = 0.030). In this subgroup, survival to hospital discharge was significantly higher in patients with transient ROSC than in those without transient ROSC (34.3% vs. 18.8%, *p* = 0.041), whereas good neurological outcome did not differ significantly between the groups (18.1% vs. 14.5%, *p* = 0.678) (Table 3). In the LFT >90-min subgroup, no significant differences in the evaluated baseline characteristics were observed according to transient ROSC status (all *p* > 0.05). Survival to hospital discharge (20.0% vs. 22.7%, *p* = 0.468) and good neurological outcome (14.8% vs. 14.4%, *p* = 0.991) were also comparable between patients with and without transient ROSC (Table 1, Table 2, Table 3 and Table 4).

#### 3.3.1. Primary Model Analysis of the Association Between Transient ROSC and Clinical Outcomes Across Low-Flow Time Categories: Prespecified (≤90 vs. >90 min) and Exploratory (<60, 60–90, and >90 min) Subgroup Analyses

In the primary analysis excluding post-arrest treatment variables, transient ROSC was associated with higher survival to hospital discharge among patients with LFT ≤90 min (adjusted OR [AOR], 2.375; 95% CI, 1.076–5.241; *p* = 0.032), whereas no significant association was observed among those with LFT >90 min (AOR, 0.884; 95% CI, 0.595–1.315; *p* = 0.543). The interaction between transient ROSC and dichotomized LFT was significant for survival to hospital discharge (*p* for interaction = 0.036). In the three-category analysis, transient ROSC was significantly associated with survival to hospital discharge in the LFT 60–90-min subgroup (AOR, 3.399; 95% CI, 1.021–11.316; *p* = 0.046), but not in the <60-min or >90-min subgroups. However, the global interaction across the three LFT categories was not statistically significant (*p* for interaction = 0.126). No significant associations between transient ROSC and good neurological outcome were observed across the LFT subgroups, and the interaction tests were not significant for either the dichotomized (*p* for interaction = 0.967) or three-category LFT classification (*p* for interaction = 0.957) (Table 5 and Figure 2).

#### 3.3.2. Sensitivity Analysis Across Low-Flow Time Categories with Additional Adjustment for Reperfusion Therapy and Targeted Temperature Management

In the sensitivity analysis with additional adjustment for reperfusion therapy and targeted temperature management, the association between transient ROSC and survival to hospital discharge remained significant in the LFT ≤90-min subgroup (AOR, 2.673; 95% CI, 1.175–6.082; *p* = 0.019), with a significant interaction between transient ROSC and dichotomized LFT (*p* for interaction = 0.011). In the three-category analysis, transient ROSC remained significantly associated with survival in the LFT 60–90-min subgroup (AOR, 4.131; 95% CI, 1.169–14.603; *p* = 0.028); in this sensitivity model, the global interaction across the three LFT categories was also significant (*p* for interaction = 0.039). No significant association or interaction was observed for good neurological outcome (Table 6).

#### 3.3.3. Sensitivity Analysis of the Association Between Transient ROSC and Clinical Outcomes According to Low-Flow Time Using a Reduced Adjustment Model

In the reduced-model sensitivity analysis, transient ROSC was significantly associated with survival to hospital discharge in the LFT ≤ 90-min subgroup (adjusted OR, 2.272; 95% CI, 1.062–4.863; *p* = 0.035), but not in the LFT > 90-min subgroup (adjusted OR, 0.884; 95% CI, 0.596–1.310; *p* = 0.538), with a significant interaction between transient ROSC and dichotomized LFT (*p* for interaction = 0.040). In the three-category analysis, no statistically significant association was observed in the <60-min (adjusted OR, 1.948; 95% CI, 0.586–6.480; *p* = 0.277), 60–90-min (adjusted OR, 2.651; 95% CI, 0.895–7.847; *p* = 0.078), or >90-min subgroup (adjusted OR, 0.884; 95% CI, 0.596–1.310; *p* = 0.538), and the global interaction was not significant (*p* for interaction = 0.137). For good neurological outcome, no significant subgroup-specific associations were observed, and the interaction was not significant using either the dichotomized (*p* for interaction = 0.932) or three-category LFT classification (*p* for interaction = 0.957) (Table S5).

## 4. Discussion

In this nationwide cohort study of patients with witnessed OHCA who underwent ECPR, transient ROSC before ECPR was not associated with survival to hospital discharge or good neurological outcome in the overall population. However, in the prespecified analysis using an LFT cutoff of 90 min, transient ROSC was associated with higher survival among patients with LFT ≤ 90 min but not among those with LFT > 90 min, with a significant interaction between transient ROSC and LFT. In the exploratory three-category analysis, the primary model showed the strongest association with survival in the 60–90-min subgroup. However, this association was accompanied by a wide confidence interval and was attenuated in the reduced-model sensitivity analysis. Furthermore, the global interaction across the three LFT categories varied according to model specification. No corresponding association or interaction was observed for good neurological outcome. Collectively, these findings suggest that the prognostic association between transient ROSC and survival may vary according to LFT, while the more granular three-category findings should be considered exploratory and interpreted cautiously.

Several mechanisms may explain the observed LFT-dependent association between transient ROSC and survival. First, transient ROSC may temporarily interrupt a prolonged period of inadequate circulation and thereby reduce cumulative ischemic burden. LFT in ECPR studies is conventionally defined as the interval from the initiation of conventional CPR to the establishment of extracorporeal circulation [3]. This chronological definition, however, does not account for periods of spontaneous circulation occurring before ECMO initiation. Consequently, two patients with an identical chronological LFT may have experienced substantially different physiological ischemic burdens depending on the occurrence, duration, and quality of transient ROSC. This concept is biologically plausible because cardiac arrest and prolonged inadequate circulation result in systemic ischemia, whereas ROSC restores more effective organ perfusion while initiating the systemic ischemia–reperfusion response characteristic of post-cardiac arrest syndrome [16]. Thus, even temporary restoration of spontaneous circulation during prolonged resuscitation may provide a period of improved systemic and cerebral perfusion and partially interrupt otherwise continuous ischemic exposure. Nevertheless, this potential mechanism cannot be directly established from the present registry data.

The prognostic relevance of transient ROSC may become more apparent as LFT increases. Previous ECPR studies have consistently demonstrated an inverse relationship between LFT and survival or good neurological outcome, supporting the cumulative adverse effects of prolonged inadequate perfusion [3,4,13,14]. In our prespecified analysis, transient ROSC was associated with survival among patients with LFT ≤ 90 min but not among those with LFT >90 min. In the exploratory analysis further stratifying LFT into <60, 60–90, and >90 min, the primary model demonstrated the association in the 60–90-min subgroup. One possible explanation is that among selected ECPR candidates with relatively short LFT, cumulative ischemic injury may be less advanced, making the additional prognostic information provided by transient ROSC more difficult to detect. As LFT becomes prolonged, temporary restoration of spontaneous circulation may become increasingly relevant by intermittently restoring organ perfusion before extracorporeal circulation is established.

This interpretation is broadly consistent with findings from the SAVE-J II registry. In an analysis restricted to patients with prolonged LFT ≥60 min, good neurological recovery remained achievable despite prolonged resuscitation, occurring in 71 of 708 patients (10.0%) [7]. Transient ROSC was independently associated with good neurological outcome in that population, together with younger age, shockable rhythm at hospital arrival, and signs of life [7]. These observations suggest that transient ROSC may provide information regarding residual physiological viability or resuscitability in selected patients despite prolonged low-flow exposure. However, our exploratory three-category findings do not establish a discrete LFT interval within which transient ROSC confers benefit and should not be interpreted as defining a therapeutic window.

Conversely, the absence of a significant association between transient ROSC and survival among patients with LFT > 90 min may reflect the dominant adverse consequences of extremely prolonged inadequate perfusion. Previous studies have demonstrated marked reductions in survival with increasing LFT. In a large nationwide ECPR study of in-hospital cardiac arrest, estimated survival to discharge decreased to 7.9% among patients with a low-flow duration > 90 min [6]. Studies of OHCA treated with ECPR have similarly demonstrated progressively poorer survival and neurological outcomes with increasing LFT [3,4,17,18]. Extended systemic ischemia and subsequent reperfusion can lead to post-cardiac arrest cerebral injury, myocardial dysfunction, systemic ischemia–reperfusion injury, and ongoing circulatory failure [13,14]. After extremely prolonged low-flow exposure, a temporary period of spontaneous circulation may therefore be insufficient to offset accumulated cellular and organ injury. Nevertheless, LFT > 90 min should not be regarded as an absolute threshold for futility because good outcomes have occasionally been reported despite markedly prolonged LFT, and LFT should be interpreted in conjunction with other prognostic characteristics [7].

An alternative but complementary explanation is that transient ROSC may serve not only as a temporary physiological interruption of ischemia but also as a marker of underlying resuscitability. Patients who achieve transient ROSC during otherwise refractory cardiac arrest may have greater cardiovascular reserve, greater reversibility of the underlying cause, or greater responsiveness to resuscitative interventions than those who never achieve spontaneous circulation. Previous ECPR studies support the prognostic relevance of ROSC before extracorporeal support. A systematic review and meta-analysis identified ROSC before ECPR as a factor associated with improved short-term survival [17,19], and an earlier ECPR cohort study reported that any ROSC event before ECPR was an independent predictor of survival [20]. More recently, transient ROSC was independently associated with good neurological outcome at 180 days among patients with refractory OHCA treated with ECPR (adjusted OR, 8.37; 95% CI, 2.20–38.79), and decision-tree analysis identified transient ROSC as an upstream factor associated with neurological recovery [21]. Taken together, these observations suggest that transient ROSC may provide complementary prognostic information beyond chronological LFT by reflecting both temporary restoration of perfusion and the underlying physiological responsiveness of the patient.

The absence of a corresponding association with good neurological outcome warrants consideration. Survival and neurological recovery may respond differently to temporary restoration of circulation. Although transient ROSC may provide sufficient systemic perfusion to support short-term circulatory viability, the duration and quality of restored cerebral perfusion may be insufficient to prevent hypoxic–ischemic brain injury. Moreover, neurological outcome is influenced by multiple factors beyond restoration of systemic circulation, including the duration and severity of cerebral ischemia and subsequent post-cardiac arrest injury. In the present study, neither the prespecified dichotomized analysis nor the exploratory three-category analysis demonstrated significant interaction for good neurological outcome. Therefore, the observed LFT-dependent association with survival should not be extrapolated to neurological recovery.

The sensitivity analyses provide additional context for interpretation of these findings. In response to the potential influence of post-arrest treatment variables, the primary model focused on characteristics available before or during resuscitation, whereas reperfusion therapy and targeted temperature management were additionally included in a sensitivity analysis. The association between transient ROSC and survival in the LFT ≤ 90-min subgroup and the interaction using the prespecified 90-min cutoff remained statistically significant after additional adjustment for these post-arrest treatments. The association in the 60–90-min subgroup also remained significant in this sensitivity model. However, the global interaction across the three LFT categories changed from non-significant in the primary model (*p* = 0.126) to significant after additional adjustment for post-arrest treatments (*p* = 0.039). This variation indicates that evidence for effect modification across the more granular three-category classification was sensitive to model specification and should therefore be interpreted cautiously.

The limited number of outcome events in the shorter LFT subgroups represents an additional concern regarding model stability. To address potential overfitting, we performed a sensitivity analysis using a reduced model containing seven clinically important covariates (Table S5). The significant interaction between transient ROSC and dichotomized LFT for survival remained in the reduced model, supporting the robustness of the finding that the association differed between patients with LFT ≤ 90 and >90 min. In contrast, the association in the 60–90-min subgroup and the global interaction across the three LFT categories were no longer statistically significant in the reduced model. Importantly, the direction of the association in the 60–90-min subgroup remained toward higher survival with transient ROSC, but the estimate was imprecise. These findings reinforce the distinction between the relatively robust prespecified dichotomized analysis and the less stable exploratory three-category analysis. Accordingly, the latter should be considered hypothesis-generating rather than confirmatory.

Mechanical CPR also warrants consideration as a potential source of confounding. In the LFT 60–90-min subgroup, mechanical CPR was more frequently used among patients with transient ROSC than among those without transient ROSC. Mechanical CPR was therefore included as a covariate in the multivariable models because its use may reflect differences in resuscitation processes and may be associated with clinical outcomes. Mechanical chest-compression devices can facilitate continuous compressions during patient transport, ECPR preparation, and cannulation; however, evidence that mechanical CPR improves clinical outcomes compared with manual CPR remains inconclusive. Mechanical CPR has also been associated with traumatic complications, including intrathoracic injury, in patients undergoing ECPR [22,23]. Thus, the imbalance in mechanical CPR use may reflect differences in resuscitation practice and remains a potential source of residual confounding despite statistical adjustment.

An important methodological consideration is that LFT in this study represented chronological rather than physiological low-flow duration. LFT was measured from CPR initiation to the establishment of extracorporeal flow, and periods of transient ROSC were not subtracted from this interval. Consequently, patients with the same recorded LFT may have experienced substantially different durations of actual low-flow circulation and cumulative ischemic burden. Because detailed information regarding the location, duration, frequency, timing, and hemodynamic quality of transient ROSC was unavailable, cumulative physiological low-flow duration could not be quantified. This limitation is particularly relevant when interpreting the subgroup and interaction analyses and precludes causal interpretation of the observed LFT-dependent associations.

This study has several limitations. First, its retrospective registry-based design is subject to selection bias, residual confounding, and misclassification, and the highly selected population of patients undergoing ECPR may limit generalizability. Second, The registry did not capture several potentially relevant physiological and laboratory measures, such as serum lactate levels and other indicators of tissue perfusion, which limited our ability to account for the underlying severity of ischemic injury. Third, LFT was measured chronologically from CPR initiation to extracorporeal flow without accounting for transient ROSC, and detailed characteristics of transient ROSC were unavailable. Thus, recorded LFT may not accurately reflect actual low-flow duration or cumulative ischemic burden, limiting causal interpretation of the observed LFT-dependent associations. Fourth, hospital identifiers and institution-specific ECPR eligibility criteria were unavailable; therefore, clustering at the hospital level and between-center variation in ECPR selection and post-cardiac arrest management could not be evaluated. Fifth, information on no-flow time was unavailable. Because no-flow duration is an important prognostic factor after OHCA, residual confounding related to unmeasured differences in no-flow time cannot be excluded. Finally, the numbers of survival and good neurological outcome events were limited in the shorter LFT subgroups, resulting in wide confidence intervals and potential model instability. Although reduced-model sensitivity analyses were performed to address potential overfitting, these analyses cannot eliminate uncertainty arising from the limited number of events. Larger studies with more outcome events and detailed characterization of transient ROSC are needed to validate these findings and better characterize the relationship between chronological LFT, physiological ischemic burden, and transient ROSC.

## 5. Conclusions

The association between transient ROSC and survival after ECPR differed according to LFT in the prespecified analysis using a 90-min cutoff, and this interaction remained significant across sensitivity analyses. In contrast, the three-category LFT analysis was less robust to model specification and should be considered exploratory. No corresponding interaction was observed for good neurological outcome. These findings suggest that transient ROSC may provide complementary prognostic information to chronological LFT in patients with witnessed OHCA undergoing ECPR.

## Figures and Tables

**Figure 1:**
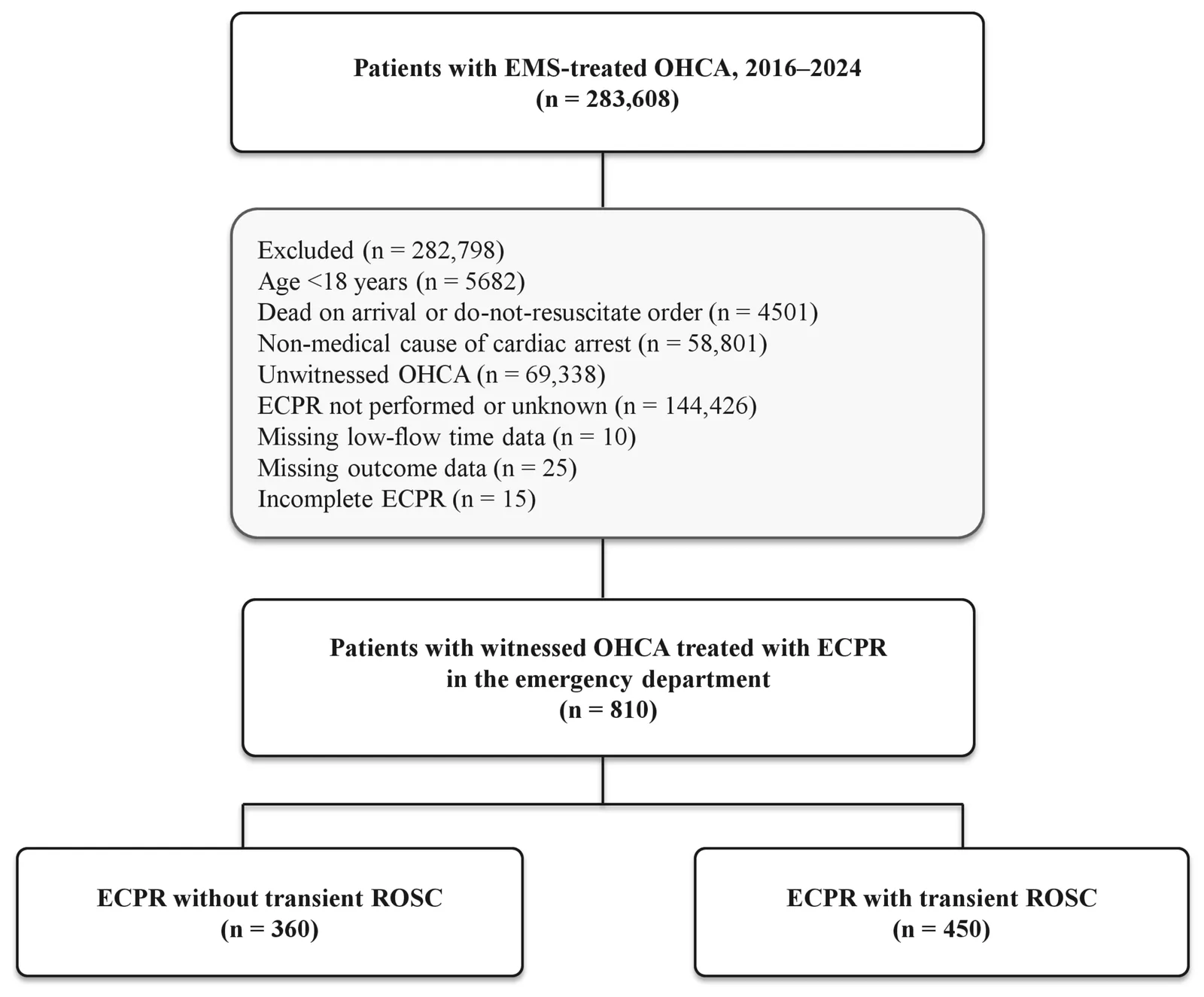
Flowchart of study population selection. EMS, emergency medical services; OHCA, out-of-hospital cardiac arrest; ECPR, extracorporeal cardiopulmonary resuscitation; ROSC, return of spontaneous circulation.

**Figure 2:**
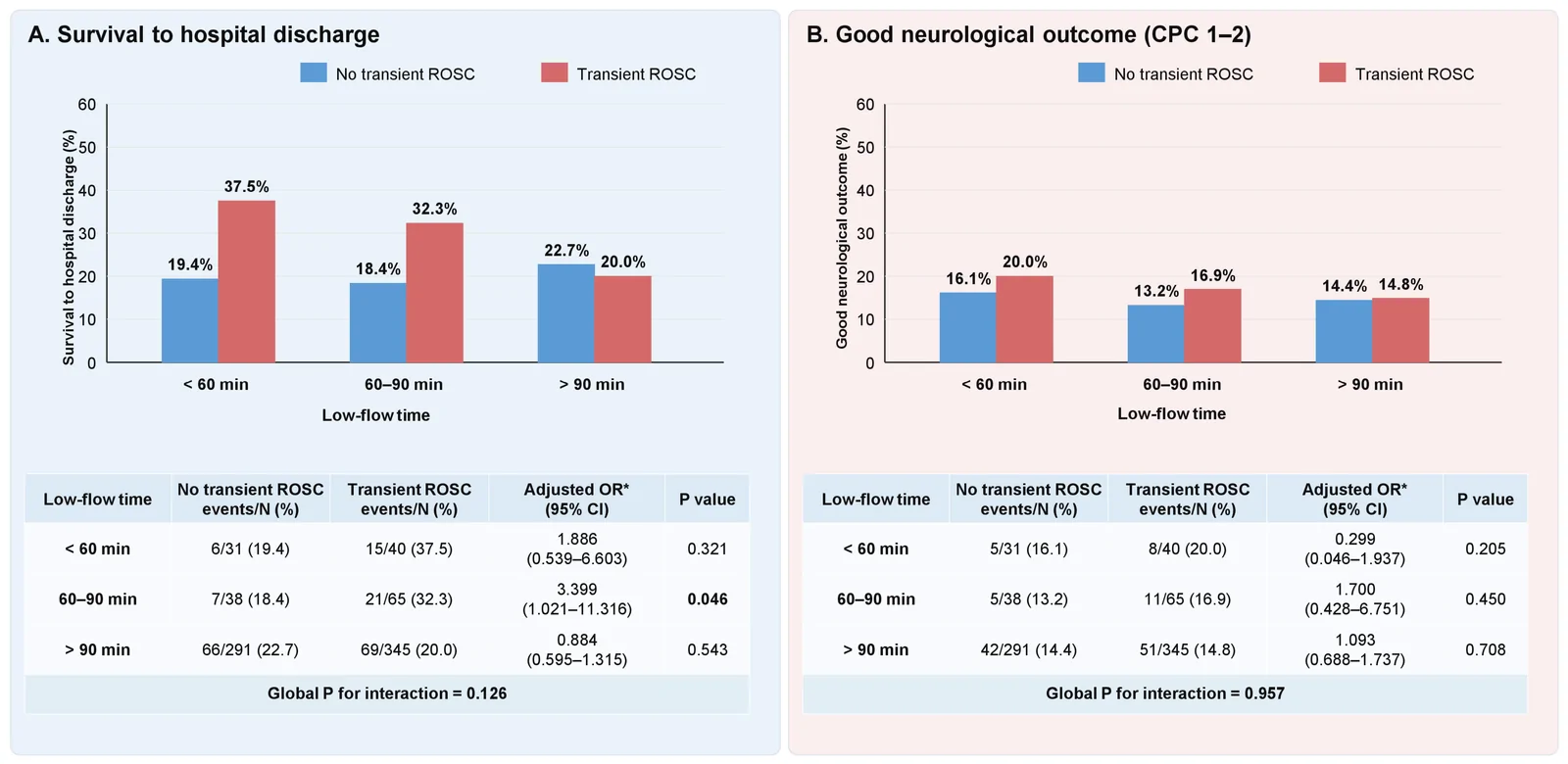
Association Between Transient ROSC and Clinical Outcomes Across Low-Flow Time Categories in Patients With Witnessed OHCA Undergoing ECPR. (**A**) Survival to hospital discharge and (**B**) good neurological outcome according to transient ROSC status across the three LFT categories (<60, 60–90, and >90 min). Abbreviations: CI, confidence interval; ECPR, extracorporeal cardiopulmonary resuscitation; LFT, low-flow time; OR, odds ratio; ROSC, return of spontaneous circulation; CPR, cardiopulmonary resuscitation. * Odds ratios were adjusted for age, sex, bystander CPR, cause of cardiac arrest, initial shockable rhythm, diabetes mellitus, hypertension, heart disease, chronic kidney disease, stroke, respiratory disease, mechanical CPR, and LFT.

**Table 1:** Patient Characteristics by Transient ROSC Status Before ECPR Initiation for LFT < 60 min.

Variables	Total	No Transient ROSC ^a^ Before ECPR	Transient ROSC ^a^ Before ECPR	*p*
	(N = 71)	(N = 31)	(N = 40)	
Sex (male), n (%)	59 (83.1%)	24 (77.4%)	35 (87.5%)	0.421
Age, years, median (IQR)	60.0 [48.0–66.5]	61.0 [50.5–68.5]	58.0 [46.0–65.5]	0.316
Bystander CPR, n (%)	16 (22.5%)	5 (16.1%)	11 (27.5%)	0.395
Cardiac cause, n (%)	69 (97.2%)	30 (96.8%)	39 (97.5%)	1.000
Initial shockable rhythm, n (%)	31 (43.7%)	10 (32.3%)	21 (52.5%)	0.143
Pre-existing comorbidity, n (%)				
Hypertension	23 (32.4%)	10 (32.3%)	13 (32.5%)	1.000
Diabetes mellitus	15 (21.1%)	7 (22.6%)	8 (20.0%)	1.000
Heart disease	12 (16.9%)	3 (9.7%)	9 (22.5%)	0.267
Chronic kidney disease	2 (2.8%)	1 (3.2%)	1 (2.5%)	1.000
Respiratory disease	3 (4.2%)	1 (3.2%)	2 (5.0%)	1.000
Stroke	1 (1.4%)	1 (3.2%)	0 (0.0%)	0.437
Mechanical CPR, n (%)	25 (35.2%)	10 (32.3%)	15 (37.5%)	0.835
Post-cardiac arrest care				
Reperfusion therapy ^b^	55 (77.5%)	23 (74.2%)	32 (80.0%)	0.768
TTM	10 (14.1%)	5 (16.1%)	5 (12.5%)	0.738
LFT, min, median (IQR)	41.0 [33.0–47.5]	44.0 [34.5–50.5]	39.0 [31.0–45.0]	0.157
Outcome at hospital discharge				
Survival discharge	21 (29.6%)	6 (19.4%)	15 (37.5%)	0.162
Good neurological outcome	13 (18.3%)	5 (16.1%)	8 (20.0%)	0.913

Abbreviations: CPR, cardiopulmonary resuscitation; ECPR, extracorporeal cardiopulmonary resuscitation; IQR, interquartile range; ROSC, return of spontaneous circulation TTM, targeted temperature management; LFT, low-flow time. Shockable rhythm was defined as ventricular fibrillation or pulseless ventricular tachycardia. LFT was defined as the chronological interval from the initiation of CPR to the establishment of extracorporeal blood flow. ^a^ Transient ROSC was considered present when spontaneous circulation was restored for >1 min but <20 min before ECPR initiation. ^b^ Reperfusion therapy comprised intravenous thrombolysis and percutaneous coronary intervention.

**Table 2:** Patient Characteristics by Transient ROSC Status Before ECPR Initiation for LFT of 60–90 min.

Variables	Total	No Transient ROSC ^a^ Before ECPR	Transient ROSC ^a^ Before ECPR	*p*
	(N = 103)	(N = 38)	(N = 65)	
Sex (male), n (%)	76 (73.8%)	29 (76.3%)	47 (72.3%)	0.830
Age, years, median (IQR)	58.0 [46.5–66.5]	57.5 [48.0–68.0]	59.0 [45.0–66.0]	0.975
Bystander CPR, n (%)	35 (34.0%)	17 (44.7%)	18 (27.7%)	0.122
Cardiac cause, n (%)	95 (92.2%)	35 (92.1%)	60 (92.3%)	1.000
Initial shockable rhythm, n (%)	36 (35.0%)	15 (39.5%)	21 (32.3%)	0.602
Pre-existing comorbidity, n (%)				
Hypertension	40 (38.8%)	13 (34.2%)	27 (41.5%)	0.598
Diabetes mellitus	25 (24.3%)	9 (23.7%)	16 (24.6%)	1.000
Heart disease	23 (22.3%)	7 (18.4%)	16 (24.6%)	0.629
Chronic kidney disease	5 (4.9%)	3 (7.9%)	2 (3.1%)	0.355
Respiratory disease	3 (2.9%)	1 (2.6%)	2 (3.1%)	1.000
Stroke	3 (2.9%)	2 (5.3%)	1 (1.5%)	0.553
Mechanical CPR, n (%)	36 (35.0%)	7 (18.4%)	29 (44.6%)	0.013
Post-cardiac arrest care				
Reperfusion therapy ^b^	74 (71.8%)	29 (76.3%)	45 (69.2%)	0.586
TTM	21 (20.4%)	8 (21.1%)	13 (20.0%)	1.000
LFT, min, median (IQR)	84.0 [78.0–88.0]	84.0 [77.0–88.0]	84.0 [78.0–88.0]	0.869
Outcome at hospital discharge				
Survival discharge	28 (27.2%)	7 (18.4%)	21 (32.3%)	0.194
Good neurological outcome	16 (15.5%)	5 (13.2%)	11 (16.9%)	0.820

Abbreviations: CPR, cardiopulmonary resuscitation; ECPR, extracorporeal cardiopulmonary resuscitation; IQR, interquartile range; ROSC, return of spontaneous circulation TTM, targeted temperature management; LFT, low-flow time. Shockable rhythm was defined as ventricular fibrillation or pulseless ventricular tachycardia. LFT was defined as the chronological interval from the initiation of CPR to the establishment of extracorporeal blood flow. ^a^ Transient ROSC was considered present when spontaneous circulation was restored for >1 min but <20 min before ECPR initiation. ^b^ Reperfusion therapy comprised intravenous thrombolysis and percutaneous coronary intervention.

**Table 3:** Patient Characteristics by Transient ROSC Status Before ECPR Initiation for LFT ≤ 90 min.

Variables	Total	No Transient ROSC ^a^ Before ECPR	Transient ROSC ^a^ Before ECPR	*p*
	(N = 174)	(N = 69)	(N = 105)	
Sex (male), n (%)	135 (77.6%)	53 (76.8%)	82 (78.1%)	0.990
Age, years, median (IQR)	59.0 [47.0–67.0]	60.0 [49.0–68.0]	59.0 [45.0–66.0]	0.423
Bystander CPR, n (%)	51 (29.3%)	22 (31.9%)	29 (27.6%)	0.664
Cardiac cause, n (%)	164 (94.3%)	65 (94.2%)	99 (94.3%)	1.000
Initial shockable rhythm, n (%)	67 (38.5%)	25 (36.2%)	42 (40.0%)	0.734
Pre-existing comorbidity, n (%)				
Hypertension	63 (36.2%)	23 (33.3%)	40 (38.1%)	0.633
Diabetes mellitus	40 (23.0%)	16 (23.2%)	24 (22.9%)	1.000
Heart disease	35 (20.1%)	10 (14.5%)	25 (23.8%)	0.191
Chronic kidney disease	7 (4.0%)	4 (5.8%)	3 (2.9%)	0.437
Respiratory disease	6 (3.4%)	2 (2.9%)	4 (3.8%)	1.000
Stroke	4 (2.3%)	3 (4.3%)	1 (1.0%)	0.302
Mechanical CPR, n (%)	61 (35.1%)	17 (24.6%)	44 (41.9%)	0.030
Post-cardiac arrest care				
Reperfusion therapy ^b^	129 (74.1%)	52 (75.4%)	77 (73.3%)	0.903
TTM	31 (17.8%)	13 (18.8%)	18 (17.1%)	0.933
LFT, min, median (IQR)	72.5 [44.0–85.0]	70.0 [44.0–85.0]	74.0 [44.0–85.0]	0.758
Outcome at hospital discharge				
Survival discharge	49 (28.2%)	13 (18.8%)	36 (34.3%)	0.041
Good neurological outcome	29 (16.7%)	10 (14.5%)	19 (18.1%)	0.678

Abbreviations: CPR, cardiopulmonary resuscitation; ECPR, extracorporeal cardiopulmonary resuscitation; IQR, interquartile range; ROSC, return of spontaneous circulation TTM, targeted temperature management; LFT, low-flow time. Shockable rhythm was defined as ventricular fibrillation or pulseless ventricular tachycardia. LFT was defined as the chronological interval from the initiation of CPR to the establishment of extracorporeal blood flow. ^a^ Transient ROSC was considered present when spontaneous circulation was restored for >1 min but <20 min before ECPR initiation. ^b^ Reperfusion therapy comprised intravenous thrombolysis and percutaneous coronary intervention.

**Table 4:** Patient Characteristics by Transient ROSC Status Before ECPR Initiation for LFT > 90 min.

Variables	Total	No Transient ROSC ^a^ Before ECPR	Transient ROSC ^a^ Before ECPR	*p*
	(N = 636)	(N = 291)	(N = 345)	
Sex (male), n (%)	539 (84.7%)	244 (83.8%)	295 (85.5%)	0.639
Age, years, median (IQR)	55.0 [45.0–64.0]	54.0 [44.5–63.0]	56.0 [46.0–64.0]	0.306
Bystander CPR, n (%)	290 (45.6%)	127 (43.6%)	163 (47.2%)	0.407
Cardiac cause, n (%)	618 (97.2%)	284 (97.6%)	334 (96.8%)	0.724
Initial shockable rhythm, n (%)	238 (37.4%)	113 (38.8%)	125 (36.2%)	0.553
Pre-existing comorbidity, n (%)				
Hypertension	232 (36.5%)	107 (36.8%)	125 (36.2%)	0.954
Diabetes mellitus	153 (24.1%)	73 (25.1%)	80 (23.2%)	0.642
Heart disease	143 (22.5%)	60 (20.6%)	83 (24.1%)	0.347
Chronic kidney disease	25 (3.9%)	9 (3.1%)	16 (4.6%)	0.427
Respiratory disease	16 (2.5%)	8 (2.7%)	8 (2.3%)	0.927
Stroke	21 (3.3%)	8 (2.7%)	13 (3.8%)	0.621
Mechanical CPR, n (%)	236 (37.1%)	115 (39.5%)	121 (35.1%)	0.283
Post-cardiac arrest care				
Reperfusion therapy ^b^	436 (68.6%)	191 (65.6%)	245 (71.0%)	0.171
TTM	116 (18.2%)	53 (18.2%)	63 (18.3%)	1.000
LFT, min, median (IQR)	117.5 [103.0–160.0]	117.0 [104.0–161.0]	118.0 [103.0–160.0]	0.535
Outcome at hospital discharge				
Survival discharge	135 (21.2%)	66 (22.7%)	69 (20.0%)	0.468
Good neurological outcome	93 (14.6%)	42 (14.4%)	51 (14.8%)	0.991

Abbreviations: CPR, cardiopulmonary resuscitation; ECPR, extracorporeal cardiopulmonary resuscitation; IQR, interquartile range; ROSC, return of spontaneous circulation TTM, targeted temperature management; LFT, low-flow time. Shockable rhythm was defined as ventricular fibrillation or pulseless ventricular tachycardia. LFT was defined as the chronological interval from the initiation of CPR to the establishment of extracorporeal blood flow. ^a^ Transient ROSC was considered present when spontaneous circulation was restored for >1 min but <20 min before ECPR initiation. ^b^ Reperfusion therapy comprised intravenous thrombolysis and percutaneous coronary intervention.

**Table 5:** Association Between Transient ROSC and Clinical Outcomes According to Low-Flow Time in the Primary Model Analysis.

LFT ≤90 min vs. >90 min						
Outcome	LFT Subgroup	No Transient ROSC, Events/N (%)	Transient ROSC, Events/N (%)	Adjusted OR * (95% CI)	*p* Value	Global *p* for Interaction
Survival to hospital discharge	≤90 min	13/69 (18.8)	36/105 (34.3)	2.375 (1.076–5.241)	0.032	0.036
	>90 min	66/291 (22.7)	69/345 (20.0)	0.884 (0.595–1.315)	0.543	
Good neurological outcome	≤90 min	10/69 (14.5)	19/105 (18.1)	1.007 (0.389–2.608)	0.988	0.967
	>90 min	42/291 (14.4)	51/345 (14.8)	1.093 (0.688–1.737)	0.708	
LFT <60, 60–90, and >90 min						
Survival to hospital discharge	<60 min	6/31 (19.4)	15/40 (37.5)	1.886 (0.539–6.603)	0.321	0.126
	60–90 min	7/38 (18.4)	21/65 (32.3)	3.399 (1.021–11.316)	0.046	
	>90 min	66/291 (22.7)	69/345 (20.0)	0.884 (0.595–1.315)	0.543	
Good neurological outcome	<60 min	5/31 (16.1)	8/40 (20.0)	0.299 (0.046–1.937)	0.205	0.957
	60–90 min	5/38 (13.2)	11/65 (16.9)	1.700 (0.428–6.751)	0.450	
	>90 min	42/291 (14.4)	51/345 (14.8)	1.093 (0.688–1.737)	0.708	

Abbreviations: CI, confidence interval; ECPR, extracorporeal cardiopulmonary resuscitation; LFT, low-flow time; OR, odds ratio; ROSC, return of spontaneous circulation; CPR, cardiopulmonary resuscitation. * Odds ratios were adjusted for age, sex, bystander CPR, cause of cardiac arrest, initial shockable rhythm, diabetes mellitus, hypertension, heart disease, chronic kidney disease, stroke, respiratory disease, mechanical CPR, and LFT.

**Table 6:** Sensitivity Analysis of the Association Between Transient ROSC and Clinical Outcomes According to Low-Flow Time With Additional Adjustment for Reperfusion Therapy and Targeted Temperature Management.

LFT ≤90 min vs. >90 min						
Outcome	LFT Subgroup	No Transient ROSC, Events/N (%)	Transient ROSC, Events/N (%)	Adjusted OR * (95% CI)	*p* Value	Global *p* for Interaction
Survival to hospital discharge	≤90 min	13/69 (18.8)	36/105 (34.3)	2.673 (1.175–6.082)	0.019	0.011
	>90 min	66/291 (22.7)	69/345 (20.0)	0.809 (0.538–1.217)	0.309	
Good neurological outcome	≤90 min	10/69 (14.5)	19/105 (18.1)	1.119 (0.410–3.052)	0.826	0.688
	>90 min	42/291 (14.4)	51/345 (14.8)	0.983 (0.611–1.579)	0.942	
LFT <60, 60–90, and >90 min						
Survival to hospital discharge	<60 min	6/31 (19.4)	15/40 (37.5)	2.277 (0.583–8.891)	0.237	0.039
	60–90 min	7/38 (18.4)	21/65 (32.3)	4.131 (1.169–14.603)	0.028	
	>90 min	66/291 (22.7)	69/345 (20.0)	0.813 (0.537–1.230)	0.328	
Good neurological outcome	<60 min	5/31 (16.1)	8/40 (20.0)	0.399 (0.053–3.014)	0.373	0.837
	60–90 min	5/38 (13.2)	11/65 (16.9)	2.336 (0.507–10.754)	0.276	
	>90 min	42/291 (14.4)	51/345 (14.8)	1.005 (0.620–1.629)	0.983	

Abbreviations: CI, confidence interval; ECPR, extracorporeal cardiopulmonary resuscitation; LFT, low-flow time; OR, odds ratio; ROSC, return of spontaneous circulation; CPR, cardiopulmonary resuscitation. * Odds ratios were adjusted for age, sex, bystander CPR, cause of cardiac arrest, initial shockable rhythm, diabetes mellitus, hypertension, heart disease, chronic kidney disease, stroke, respiratory disease, mechanical CPR, and LFT, targeted temperature management, and reperfusion therapy.

## Data Availability

The authors used a database made available by the Korea Disease Control and Prevention Agency, which holds authority over the OHCA registry dataset in Korea. Access to this dataset requires permission and interested parties can request access through the official website (https://www.kdca.go.kr/injury/biz/injury/main/mainPage.do) (accessed on 10 April 2026).

## Supplementary Materials

The following supporting information can be downloaded at: https://www.mdpi.com/article/10.3390/jcm15197354/s1, Table S1: Definition and detailed classification of pre-existing comorbidity; Table S2: Glasgow-Pittsburgh Cerebral Performance Categories scores and their corresponding descriptions; Table S3: Characteristics of the study population by transient ROSC status before ECPR initiation at ED; Table S4: Analysis of survival and neurological outcomes using multivariable logistic regression.; Table S5: Sensitivity Analysis of the Association Between Transient ROSC and Clinical Outcomes According to Low-Flow Time Using a Reduced Adjustment Model; Table S6: Missing data for variables; STROBE Checklist: Strengthening the Reporting of Observational Studies in Epidemiology (STROBE) checklist.

## Abbreviations

The following abbreviations are used in this manuscript:

CPC	Cerebral Performance Category
CPR	Cardiopulmonary resuscitation
ECPR	Extracorporeal cardiopulmonary resuscitation
LFT	Low-flow time
ED	Emergency department
EMS	Emergency medical services
IRB	Institutional Review Board
KCDC	Korea Centers for Disease Control and Prevention
OHCA	out-of-hospital cardiac arrest
OHCAS	Out-of-Hospital Cardiac Arrest Surveillance
PCI	Percutaneous coronary intervention
ROSC	Return of spontaneous circulation
TTM	Targeted temperature management
