# Supplementary material for: Prognostic Impact of Transient Return of Spontaneous Circulation According to Low-Flow Time in Patients with Out-of-Hospital Cardiac Arrest Undergoing Extracorporeal Cardiopulmonary Resuscitation: A Nationwide Cohort Study
Source: J Clin Med. 2026 Sep 22;15(19):7354. doi: 10.3390/jcm15197354 (PMC13666486; doi:10.3390/jcm15197354)
Supplement: Supplementary file 1 [file jcm-15-07354-s001.zip › Supplementary materials_Transient ROSC Before ECPR_LFT_revised_final.pdf]

Table S1. Definition and detailed classification of pre-existing comorbidity

Disease	Definition and detailed classification
Hypertension	The disease was diagnosed by the doctor prior to cardiac arrest and is clearly stated in the medical record
Diabetes mellitus	The disease was diagnosed by the doctor prior to cardiac arrest and is clearly stated in the medical record.
Heart disease	The disease was diagnosed by the doctor prior to cardiac arrest and is clearly stated in the medical record.<br>(Ischemic heart disease, myocardial infarction, angina, valvular heart disease, arrhythmia, congestive heart disease, myocardiopathy, congenital heart disease, previous heart intervention record including stent, primary coronary intervention, coronary artery bypass grafting, and other heart disease)
Chronic renal disease	The disease was diagnosed by the doctor prior to cardiac arrest and is clearly stated in the medical record.<br>(chronic renal failure, dialysis, kidney transplantation, and other chronic kidney disease)
Chronic respiratory disease	The disease was diagnosed by the doctor prior to cardiac arrest and is clearly stated in the medical record. (Asthma, chronic obstructive pulmonary disease, pulmonary tuberculosis, other chronic respiratory disease.)
Stroke	The disease was diagnosed by the doctor prior to cardiac arrest and is clearly stated in the medical record.<br>(cerebral infarction, cerebral haemorrhage, and unclassified stroke)
Dyslipidemia	The disease was diagnosed by the doctor prior to cardiac arrest and is clearly stated in the medical record.<br>(hyperlipidaemia and unclassified dyslipidemia)

Table S2. Glasgow-Pittsburgh Cerebral Performance Categories scores and their corresponding descriptions

CPC Score	Description
CPC 1	Good cerebral performance with mild or no neurological deficit
CPC 2	Moderate cerebral disability with moderate neurological deficit, sufficient functional independence for activities of daily living
CPC 3	Severe cerebral disability with severe neurological deficit, dependence on others for daily activities
CPC 4	Coma or vegetative state, absence of meaningful neurological function, inability to interact with the environment
CPC 5	Brain death or death due to neurological injury

Abbreviations: CPC, Cerebral Performance Category.

Table S3. Characteristics of the study population by transient ROSC status before ECPR initiation at ED.

Variables	Total (N=810)	No transient ROSC [1] before ECPR (N=360)	Transient ROSC [1] before ECPR (N=450)	p
Sex (male), n (%)	674 (83.2%)	297 (82.5%)	377 (83.8%)	0.697
Age, years, median (IQR)	56.0 [45.0-64.0]	55.0 [45.0-64.0]	57.0 [46.0-65.0]	0.541
Bystander CPR, n (%)	341 (42.1%)	149 (41.4%)	192 (42.7%)	0.657
Cardiac cause, n (%)	782 (96.5%)	349 (96.9%)	433 (96.2%)	0.570
Initial shockable rhythm, n (%)	305 (37.7%)	138 (38.3%)	167 (37.1%)	0.777
Pre-existing comorbidity, n (%)				
Hypertension	295 (36.4%)	130 (36.1%)	165 (36.7%)	0.928
Diabetes mellitus	193 (23.8%)	89 (24.7%)	104 (23.1%)	0.651
Heart disease	178 (22.0%)	70 (19.4%)	108 (24.0%)	0.141
Chronic kidney disease	32 (4.0%)	13 (3.6%)	19 (4.2%)	0.793
Respiratory disease	22 (2.7%)	10 (2.8%)	12 (2.7%)	1.000
Stroke	25 (3.1%)	11 (3.1%)	14 (3.1%)	1.000
Mechanical CPR, n (%)	297 (36.7%)	132 (36.7%)	165 (36.7%)	1.000
Post-cardiac arrest care				
Reperfusion therapy [2]	565 (69.8%)	243 (67.5%)	322 (71.6%)	0.241
TTM	147 (18.1%)	66 (18.3%)	81 (18.0%)	0.976
LFT, min, median (IQR)	108.5 [94.0-148.0]	110.0 [95.0-153.5]	108.0 [91.0-140.0]	0.155
Outcome at hospital discharge				
Survival discharge	184 (22.7%)	79 (21.9%)	105 (23.3%)	0.701
Good neurological outcome	122 (15.1%)	52 (14.4%)	70 (15.6%)	0.733

Abbreviations: CPR, cardiopulmonary resuscitation; ECPR, extracorporeal cardiopulmonary resuscitation; IQR, interquartile range; ROSC, return of spontaneous circulation, ED, emergency department; TTM, targeted temperature management; LFT, low-flow time.

Shockable rhythm was defined as ventricular fibrillation or pulseless ventricular tachycardia.

LFT was defined as the interval from CPR initiation to ECMO flow establishment; transient ROSC was not considered termination.

1 Transient ROSC was defined as a return of spontaneous circulation lasting >1 minute but <20 minutes prior to ECPR initiation.

2 Reperfusion therapy included intravenous thrombolysis and percutaneous coronary intervention.

Table S4. Analysis of survival and neurological outcomes using multivariable logistic regression.

	AOR (95%CI)	P
Survival to hospital discharge *		
Transient ROSC (-)	1	
Transient ROSC (+)	1.025(0.717-1.464)	0.892
Good neurological outcome *		
Transient ROSC (-)	1	
Transient ROSC (+)	1.016(0.668-1.544)	0.941

LFT, low-flow time; ROSC, return of spontaneous circulation; AOR, adjusted odds ratio; CI, confidence interval.
*Adjusted for age, sex, bystander CPR, presumed cardiac aetiology, initial shockable rhythm, hypertension, diabetes mellitus, heart disease, chronic kidney disease, stroke, respiratory disease, mechanical CPR, reperfusion therapy, targeted temperature management, and low-flow time.

Table S5. Sensitivity Analysis of the Association Between Transient ROSC and Clinical Outcomes According to Low-Flow Time Using a Reduced Adjustment Model

LFT ≤90 min vs. >90 min						
**Outcome**	**LFT subgroup**	**No transient ROSC, events/N (%)**	**Transient ROSC, events/N (%)**	**Adjusted OR* (95% CI)**	**P value**	**Global P for interaction**
Survival to hospital discharge	≤90 min	13/69 (18.8)	36/105 (34.3)	2.272 (1.062–4.863)	0.035	0.040
	>90 min	66/291 (22.7)	69/345 (20.0)	0.884 (0.596–1.310)	0.538	
Good neurological outcome	≤90 min	10/69 (14.5)	19/105 (18.1)	1.264 (0.522–3.061)	0.603	0.932
	>90 min	42/291 (14.4)	51/345 (14.8)	1.099 (0.693–1.742)	0.689	
**LFT <60, 60–90, and >90 min**						
Survival to hospital discharge	<60 min	6/31 (19.4)	15/40 (37.5)	1.948 (0.586–6.480)	0.277	0.137
	60–90 min	7/38 (18.4)	21/65 (32.3)	2.651 (0.895–7.847)	0.078	
	>90 min	66/291 (22.7)	69/345 (20.0)	0.884 (0.596–1.310)	0.538	
Good neurological outcome	<60 min	5/31 (16.1)	8/40 (20.0)	0.789 (0.195–3.186)	0.739	0.957
	60–90 min	5/38 (13.2)	11/65 (16.9)	1.607 (0.462–5.594)	0.456	
	>90 min	42/291 (14.4)	51/345 (14.8)	1.099 (0.693–1.742)	0.689	

Abbreviations: CI, confidence interval; ECPR, extracorporeal cardiopulmonary resuscitation; LFT, low-flow time; OR, odds ratio; ROSC, return of spontaneous circulation.

* Odds ratios were adjusted for age, sex, bystander CPR, cause of cardiac arrest, initial shockable rhythm, mechanical cardiopulmonary resuscitation, and LFT

P for interaction was derived from multivariable logistic regression models including interaction terms between transient ROSC and LFT category. For the three-category LFT analysis, the overall interaction was assessed by jointly testing the interaction terms using a likelihood-ratio test. All prespecified covariates were simultaneously entered into the models.

Table S6. Missing data for variables

**Variable**	**Missing data, n (%)***
Age	0 (0.0)
Sex	0 (0.0)
Bystander CPR	0 (0.0)
Cardiac cause	0 (0.0)
Initial shockable rhythm	0 (0.0)
Hypertension	0 (0.0)
Diabetes mellitus	0 (0.0)
Heart disease	0 (0.0)
Chronic kidney disease	0 (0.0)
Respiratory disease	0 (0.0)
Stroke	0 (0.0)
Mechanical CPR	0 (0.0)
Low-flow time	10 (1.2)
Transient ROSC before ECPR	0 (0.0)
Reperfusion therapy	0 (0.0)
Targeted temperature management	0 (0.0)
Survival to hospital discharge	12 (1.5)
Good neurological outcome at hospital discharge	13 (1.6)

* Patients with missing low-flow time data (n = 10, 1.2%) or missing outcome data (n = 25, 3.1%) were excluded before the final analytic cohort was established. Percentages are calculated using the final analytic cohort as the denominator (N = 810).

Abbreviations: CPR, cardiopulmonary resuscitation; ECPR, extracorporeal cardiopulmonary resuscitation; ROSC, return of spontaneous circulation
